# How are you coping? Stress, coping, burnout, and aggression in forensic mental healthcare workers

**DOI:** 10.3389/fpsyg.2023.1301878

**Published:** 2024-01-11

**Authors:** Pascalle Spaan, Frank van den Boogert, Yvonne H. A. Bouman, Witte J. G. Hoogendijk, Sabine J. Roza

**Affiliations:** ^1^Department of Psychiatry, Erasmus University Medical Center, Rotterdam, Netherlands; ^2^Department of Research, Transfore, Deventer, Netherlands; ^3^Science and Treatment Innovation, Fivoor, Rotterdam, Netherlands; ^4^Netherlands Institute for Forensic Psychiatry and Psychology, Utrecht, Netherlands

**Keywords:** aggression, burnout, coping behavior, healthcare workers, stress

## Abstract

**Introduction:**

Perceived stress at work has been linked to several adverse outcomes in workers, including increased risk of burnout and aggression (e.g., anger and irritability). However, much remains unknown about factors that might mitigate the negative influences of perceived stress on workers’ well-being. This study focusses on coping as a possible protective factor against perceived stress and its consequences in forensic mental healthcare workers. We aimed to identify which higher-order coping factors were present in this worker sample and to investigate whether these coping factors modify the associations between perceived stress and burnout or aggression.

**Methods:**

For this observational survey study, 116 forensic mental healthcare workers completed questionnaires assessing changes in work situation since the start of COVID-19, perceived stress, coping, burnout symptoms, and aggression.

**Results:**

Results from principal component analysis indicated that four higher-order coping factors could be distinguished: social support and emotional coping, positive cognitive restructuring, problem-focused coping, and passive coping. Higher perceived stress levels were associated with higher levels of both burnout and aggression in workers. Problem-focused coping was associated with less burnout symptoms in workers. Furthermore, positive cognitive restructuring was associated with less aggression in workers.

**Discussion:**

In conclusion, problem-focused coping and positive cognitive restructuring may protect workers against burnout symptoms and aggression and these results may inform future studies on preventive interventions aimed at promoting worker’s well-being.

## 1 Introduction

“When the going gets tough, the tough get going” is a well-known phrase that describes how some individuals work extra hard in circumstances perceived as particularly tough or stressful. The COVID-19 pandemic was such a stressful period for many workers, and has been linked to increases in perceived stress, burnout, and emotional reactions, such as anger and irritability, in workers, especially those working in healthcare professions (Giusti et al., 2020; Hacimusalar et al., 2020; Denning et al., 2021). Perceived stress is the way in which an individual perceives situations in one’s life as stressful (Cohen et al., 1983). Burnout can be defined as “a work-related state of exhaustion that occurs among employees, which is characterized by extreme tiredness, reduced ability to regulate cognitive and emotional processes, and mental distancing” (Schaufeli et al., 2020, p. 4). Schaufeli et al. (2020) further note that burnout is often accompanied by depressed mood, and non-specific psychological- and psychosomatic complaints. The effects of perceived stress on healthcare professionals, likely exacerbated by the pandemic, are concerning in a population of workers that pre-pandemic was already at high risk of adverse stress-related outcomes like burnout (Shanafelt et al., 2019; Woo et al., 2020). In fact, studies conducted during the COVID-19 pandemic demonstrate the heightened levels of burnout symptomatology among healthcare professionals and highlight the importance of enhancing resilience and acquiring effective coping strategies, such as task-oriented problem management (Di Monte et al., 2020; Di Trani et al., 2023). Moreover, previous stressful events are found to be predictive for resilience and distress in mental health professionals (Minelli et al., 2022) Mechanisms underlying the association between perceived stress and workers’ well-being, and ways to protect workers from the sequelae of stress, warrant further study.

There are several biopsychosocial theoretical frameworks that may explain the adverse outcomes that stress causes workers. First, in terms of biological mechanisms, levels of the corticosteroid cortisol increase in the body as a response to stressors. This mechanism is adaptive in the short term, as it readies an individual for dealing with the stressor by activating the sympathetic nervous system, but maladaptive when stress is chronic (e.g., Sapolsky, 2004). High cortisol concentrations in hair have been proposed as a biomarker, for chronic stress and burnout in community and worker populations (Penz et al., 2018). HCC were indeed increased in healthcare workers during COVID-19 (Ibar et al., 2021; Marcil et al., 2022), especially in workers with burnout and workers with direct patient contact (Ibar et al., 2021).

Considering psychological and social theories, several propose a similar framework where stress leads to adverse effects. The demands-resources theory hypothesizes that burnout occurs when there is an imbalance between the demands and resources derived from work (Bakker and Demerouti, 2014). Demands are factors that cost mental energy, while resources reduce costs in mental energy. The COVID-19 pandemic may have added demands, such as strict rules about distance and hygiene, and likely reduced resources, for example by not allowing workers to be physically present in the workplace. The general strain theory (GST) poses that strains or stressors increase the likelihood of negative emotions like anger and frustration (Agnew, 1992). GST originally was used as a framework to predict crime, presented as a coping strategy, in relation to these negative emotions. Stressor or strain are thought to arise in situations where personal goals are thwarted, positive stimuli are removed, or negative stimuli are added. For example, during COVID-19 workers may have been thwarted from fulfilling professional or personal goals because educational activities were canceled. Note that in the removal of positive and addition of negative stimuli, GST is similar to a demands and resources perspective about potential origins of stress. Both models predict that workers experience higher levels of stress during the COVID-19 pandemic, and therefore are at increased risk of stress-related adverse outcomes.

In addition to burnout, aggression is another potential negative outcome of stress. It is often measured in terms of anger, hostility, verbal, and physical aggression (Buss and Perry, 1992). Aggression in the workplace is an important factor of study in relation to occupational health, as it is related to various adverse organizational and health outcomes in workers, such as higher turnover intent, psychological distress, emotional exhaustion, depression, lower job satisfaction, affective commitment, and physical wellbeing (Hershcovis and Barling, 2010). Although the research is scarce, studies in various worker populations have reported that those with more burnout symptoms also experienced more aggression; this was found in populations of college students (Oreizi-Esfahani and Tomlinson, 2018), medical interns (Sahraeian and Edrisi, 2020), and policemen (Queiros et al., 2013). For example, a study in male police officers found that burnout symptoms of emotional exhaustion were positively associated with anger and hostility, symptoms of depersonalization were positively associated with overall aggression and verbal aggression, and symptoms of low personal accomplishment were positively association with physical aggression and anger (Queiros et al., 2013). It is important to note here that on average, these policemen showed levels of overall aggression that fell within the normal range, as one might expect in a non-clinical worker sample. Another reason to study aggression in workers is that it may also negatively influence the workplace atmosphere and co-workers. In a meta-analysis on various worker samples, both supervisor and co-worker aggression had stronger adverse effects than outsider aggression on physical well-being (Hershcovis and Barling, 2010). These findings are especially interesting in relation to (mental) healthcare workers, for whom studies on workplace aggression often focus on outsider aggression, from patients. In short, aggression in workers warrants further study.

In order to find ways to mitigate the adverse effects of stress on workers’ well-being, it is important to study strategies used to handle stress and how these influence stress-related outcomes. Coping can be defined as how one deals with stress (Carver, 1997). The most commonly used coping measurement is the Brief COPE (Carver, 1997; Kato, 2015). It distinguishes 14 different coping strategies: active coping, planning, suppression of competing activities, restraint, use of instrumental social support, use of emotional social support, positive reframing, acceptance, religion, venting, denial, behavioral disengagement, self-distraction, and humor. Although coping studies date back decades, there is as of yet no consensus on how these coping strategies can be organized into higher-order coping categories (Skinner et al., 2003; Folkman and Moskowitz, 2004; Compas et al., 2017). For example, Endler and Parker (1990) distinguished three higher-order categories of coping: active problem-focused coping, emotion-oriented coping, and avoidant coping, and these factors are still commonly used in research. In contrast, Carver cautioned against categorizing based on the higher-order factors found in data-sets of others and encourages authors to analyses their own dataset for factors (Carver et al., 1989) or to rely on the lower-order coping strategies. A systematic review concludes that five core categories are most commonly found in the literature: problem-focused, support seeking, avoidance, distraction, and positive cognitive restructuring (Skinner et al., 2003). These are highly similar to the four originally found by Carver et al. (1989), which included problem-focused coping, positive cognitive restructuring, combined support seeking and emotion-focused coping, and lastly a combined factor of avoidance and distraction. Although coping is a staple in research on stress, health, and well-being, further investigation into the higher-order factors of coping is warranted.

A meta-analysis study on coping in workers found that more problem-focused coping was related to less burnout symptoms and emotion-focused coping was related to more burnout symptoms (Shin et al., 2014). Some of these associations were particularly strong in nurses, compared to teachers and service employees. Studies on the role of coping in relation to stress and stress-related outcomes during COVID-19 specifically, found that adults who used more emotion-focused coping experienced more depressive symptoms (Mariani et al., 2020). In Chinese college students, coping partially mediated the positive associations between COVID-19 stressors and aggression (Hu and Sun, 2023). Active coping (‘approach strategy’) was related to less aggression and avoidance, and self-punishment coping was related to more aggression in students. In Australian adults, positive reframing, acceptance, and humor were related to better mental health, while self-blame, venting, behavioral disengagement, and self-distraction were associated with poorer mental health during COVID-19 (Gurvich et al., 2021). Rogowska et al. (2021) studied changes over time during COVID-19 and found that stress, emotion-oriented, and avoidance-oriented coping styles increased in Polish university students, whereas life satisfaction and task-oriented coping decreased. All three coping styles investigated in that study partially mediated the relationship between perceived stress and life satisfaction; with task-oriented and avoidance-oriented coping positively related to, and emotion-oriented coping negatively related to life-satisfaction. In sum, there is some evidence of more problem-focused, active coping, and positive cognitive strategies as protective factors against adverse stress-related outcomes and emotion-focused coping as a risk factor, while avoidant coping has so far shown mixed results as a coping strategy during COVID-19. A recent systematic review, in which results of 15 studies conducted during the COVID-19 pandemic were included, demonstrated how adaptive and task-oriented coping are protective for developing burnout, where maladaptive and avoidance-oriented coping are found to be predictive for burnout. The association of emotion-oriented coping with burnout is expected to be moderated by other factors, e.g., gender (Rossi et al., 2023). The effectiveness of a particular coping factor may depend on the context (Lazarus and Folkman, 1984). For example, it may be more effective to use problem-focused coping when dealing with controllable stressors, while that coping style may be maladaptive when dealing with uncontrollable stressors.

There is limited knowledge of stress, coping, burnout, and aggression in the specialized group of forensic mental healthcare professionals, that is those who work with individuals with both a mental disorder and problems with aggressive or criminal behavior. Some studies have described the large and fast changes in forensic mental healthcare during the COVID-19 pandemic. For example, workers had to implement procedures to lower the risk of infection for both themselves and for patients by means of hand hygiene, mask wearing, social distance keeping, new isolation or quarantine measures, and implement new ways of working such as remote working, increased telepsychiatry use (e.g., voice calls, videoconferencing and e-health), and decreased contact frequency with patients (Wasser et al., 2020; Foye et al., 2021; Kennedy et al., 2021). These challenges were framed as an addition to an already highly stressful work setting with a challenging patient group, where attention to both mental health and the criminal justice concerns must be weighed constantly (Wasser et al., 2020). In Dutch forensic mental healthcare workers, fear associated with the COVID-19 pandemic and work-related stress were negatively associated with workers’ psychological well-being, while resilience was positively related to well-being (Bogaerts et al., 2021).

The COVID-19 crisis provided a rare opportunity to study stress, coping, burnout, and aggression during times of a potentially increased number of, mostly uncontrollable, stressors, both at home and at work. The current study used data from Dutch workers at a larger mental healthcare organization, collected during the first months of the global COVID-19 pandemic. In a previous manuscript, we described that sensory processing difficulties were related to stress and burnout in this sample and concluded that changes in sensory stimuli may be a way to influence stress and burnout symptoms in workers (van den Boogert et al., 2022). The present study’s primary main aim was to investigate whether coping modifies the associations between perceived stress and burnout and/or aggression outcomes in forensic mental healthcare workers. Based on previous literature, we hypothesized that more perceived stress would be associated with more symptoms of burnout as well as aggression. As a secondary aim, we investigated which higher-order coping factors could be distinguished from Brief COPE data in this worker sample. We expected that persons who used problem-focused, active coping, and positive cognitive strategies would report less burnout and aggression symptoms, whereas emotion-focused coping would be associated with more symptoms of burnout and aggression. We hypothesized that at least one of the protective higher-order coping factors would modify the effect of stress on the outcomes. By studying the well-being of workers and their ways of coping with stress, we aimed to provide new insights on ways to promote their well-being.

## 2 Materials and methods

### 2.1 Study design

The current study used data from the OostWest Project, an observational survey study in a worker population. Employees aged 18 years or older of the Dimence Group Mental Health Care Institutions in The Netherlands who were willing to provide written informed consent were asked to participate. Participants who had insufficient knowledge of the Dutch language to participate in this survey study were excluded. A total of 251 employees received our study information sheet, in which the study objectives and procedures were explained, and were invited to participate. Of these, 116 employees (46%) participated in the available time window. Participants completed surveys online between June and August 2020. Upon completion of the surveys, participants received a small monetary reward. The study was approved by the institutional review board of Dimence Group (CWO-062020PSFB).

During the sample selection period, the Dutch government was slowly lifting earlier lockdown restrictions. However, the population was still advised to: avoid busy places, wash hands often and thoroughly, keep at least 1.5 meters distance, work from home, only use public transport for essential journeys, wear face masks in public transportation (required) and public indoor spaces (strongly advised), and stay home and get tested if they experienced any symptoms of COVID-19 (Government of the Netherlands, 2020). Over the course of the study some public places were allowed to reopen, taking into account the 1.5-meter distance rule for visitors, e.g., schools, museums, restaurants, and gyms. In the (forensic) mental healthcare institutions, telepsychiatry was used when feasible. Face-to-face appointments were allowed, keeping 1.5 meters distance, and following hygiene rules.

### 2.2 Measurements

#### 2.2.1 Demographics

Participants reported on their sex, age, and educational level. Educational level was based on the highest obtained diploma and originally categorized in three levels: primary school or secondary pre-vocational training; corresponding to up to 12 years of education, vocational training, secondary general, or pre-university education; corresponding to about 13–16 years of education, and higher or academic education; corresponding to over 16 years of education. Workers were also asked about their job function, years with the company and work hours per week. In terms of job function, healthcare professionals and other professionals were considered.

#### 2.2.2 Changes in work situation

We created a 15-item questionnaire to measure participants’ perceived changes in work situation since the start of the COVID-19 crisis in The Netherlands, in March 2020. Participants were asked to what extent certain factors had changed for them since the start of the crisis. We chose factors based on literature reviews of work-related risk factors for stress and burnout (Schaufeli and Bakker, 2007; López-López et al., 2019), and on changes inside this particular organization, which resulted in the following 15 items: *workload, work-life conflict, social support from colleagues, feedback from colleagues or supervisors, autonomy, involvement in decision-making, perceived appreciation at work, pleasure at work, job satisfaction, perceived job security, perceived financial security, effectiveness, experiences of aggressive incidents, ability to work safely with clients’ personal data,* and *software problems.* Answers ranged from “strongly decreased” to “strongly increased” on a 5-point scale. To quantify the total amount of change, item scores were averaged after treating both ends of the scale equally as heightened scores: “strongly decreased” (2), “somewhat decreased” (1), “unchanged” (0), “somewhat increased” (1), “strongly increased” (2). The resulting level of experienced change scores therefore ranged from 0 to 2; with higher scores indicating more perceived changes in the work situation. The amount of change score had an acceptable internal consistency in the current sample, Cronbach’s *α* = 0.74.

#### 2.2.3 Stress

The widely used 10-item version of the Perceived Stress Scale (PSS-10; Cohen et al., 1983) was used to assess perceived stress levels in the previous month. For example, participants were asked: “In the last month, how often have you felt nervous and stressed?” Participants responded on a 5-point Likert scale from “never” (0) to “very often” (4). A sum score was calculated after reverse coding the positively stated items; with higher scores indicating higher perceived stress. A systematic review reported acceptable psychometric properties for the PSS-10 in terms of internal consistency and concurrent validity (Lee, 2012). In our sample, the PSS-10 had acceptable internal consistency, Cronbach’s *α* = 0.87.

#### 2.2.4 Coping

The Dutch version (“COPE-Easy”) of the Brief COPE was used to assess ways of coping (Carver, 1997; Kleijn et al., 2000). Participants were asked how they confront difficult or stressful events in their life (e.g., “I usually get help and advice from other people” and “I usually look for something good in what is happening”) and answered on a 4-point scale ranging from “I do not do this at all” (1) to “I usually do this a lot” (4). The Brief COPE contains 28 items in total, with 14 subscales of two items. The 14 subscales mentioned in the introduction were used. The Dutch Brief COPE was found to have adequate internal consistency (Kleijn et al., 2000).

#### 2.2.5 Aggression

The Dutch version of the Aggression Questionnaire – Short Form (AQ-SF) was used to assess aggression (Buss and Perry, 1992; Hornsveld et al., 2009). An example of an item was: “Sometimes I fly off the handle for no good reason.” Participants responded on a 5-point Likert scale from “entirely disagree” (1) to “entirely agree” (5). Four subscale scores were calculated, based on summing 3 corresponding items: *physical aggression, verbal aggression, anger,* and *hostility*. In addition, a total aggression score was derived from summing the subscales. Higher scores were indicative of more aggression. The Dutch AQ-SF has shown good test–retest reliability and adequate concurrent validity with other aggression and personality measures (Hornsveld et al., 2009). However, the physical aggression scale showed floor effects (*Mdn* = 3, *IQR* = 0), with only 11 individuals scoring above the minimum scale score of 3, and the scale was removed from further analyses. In our sample, internal consistency was good, Cronbach’s *α* = 0.80.

#### 2.2.6 Burnout symptoms

The Burnout Assessment Tool (BAT) was used to assess burnout symptoms (Schaufeli et al., 2020). It contains 33 items about individuals’ experience and feelings regarding work, for example one item states: “At work, I feel mentally exhausted.” Participants were asked to rate how often the items were applicable to them on a 5-point Likert scale from “never” (Agnew, 1992) to “always” (Carver, 1997). Four core and one secondary dimension were measured, respectively: *exhaustion, mental distance, cognitive impairment, emotional impairment,* and *secondary symptoms*. The *secondary symptoms* scale measures depressed mood, psychological distress, and psychosomatic complaints. For each dimension a mean item score was calculated, with higher scores indicating more burnout symptoms. A total score was derived from averaging all core symptom scale scores. The BAT has shown adequate reliability, and convergent as well as discriminant validity with other well-being and burnout measures (Schaufeli et al., 2020). Within our study sample internal consistency was good, Cronbach’s *α* = 0.94.

### 2.3 Analytic strategy

First, a principal component analysis (PCA) with orthogonal rotation (varimax) was conducted to investigate the higher-order factorial structure of the Brief COPE in this Dutch worker sample. The first model included all 14 subscales and sampling adequacy was present, Kaiser-Meyer-Olkin (KMO) =0.61, Bartlett’s test of sphericity (91) = 348.93, *p* < 0.001. The anti-image correlation matrix showed that all subscales except for religion (*r* = 0.232) had values above 0.5 on the diagonal; religion thus showed insufficient correlation with the other subscale variables. Five factors had an eigenvalue greater than one; these factors explained 64.27% of the variance in the coping subscales. The first four factors were made up of various subscales with rotated component loadings above 0.4 and were interpretable. The fifth factor included only the religion subscale. We decided to run a second PCA model without the religion subscale, because of its low anti-image correlation diagonal value and because we did not consider a factor based on one subscale alone to represent a higher-order coping factor. In this second model, sampling adequacy was present and the anti-image correlation matrix showed only values above 0.5 on the diagonal, KMO = 0.65, Bartlett’s test of sphericity (78) = 327.19, *p* < 0.001. Four factors had an eigenvalue greater than one and explained 60.43% of coping variance.

Differences in descriptives based on worker type and sex were analyzed with *χ^2^*-tests for categorical variables, *t*-tests for normally distributed continuous variables and Mann–Whitney *U*-tests for skewed continuous variables. In order to test which work situation factors workers reported to have increased or decreased, on average, indicated by a score above or below 3, we conducted one-sample *t*-tests on the original 15 items.

For the main analyses, hierarchical linear regression models were run with covariates in the first step, main effects of stress and coping in the second step, and interaction effects between stress and coping in the third step. These last two steps were based on centered predictors. Sex, age, educational level, and healthcare professional status were used as covariates. Dependent variables were the burnout total and subscale scores. The burnout emotional impairment outcome was log-transformed in order to meet the linear regression model homoscedasticity assumption. Effect sizes of the predictor variables are expressed in Cohen’s *f^2^*, with values of 0.02 considered small, 0.15 considered medium, and.35 considered large (Cohen, 2013). For interaction effects, post-hoc simple slope analyses were conducted with the PROCESS macro, model 1 was used with the same variables as in the hierarchical linear regression (Hayes, 2022). Hierarchical binary logistic regressions were run similarly. Aggression outcomes violated linear regression model assumptions even when log, square root, or cube root transformed, and were therefore dichotomized based on the 80th percentile into lower aggression and higher aggression. According to Cohen’s (2013) guidelines, we considered an Odds Ratio (OR) to be very small when below 1.44, small when between 1.44 and 2.48, medium between 2.48 and 4.27, and large above 4.27. Finally, sensitivity analyses were run to investigate the potential interaction effects of sex, education level, and healthcare professional status with stress on the burnout and aggression outcomes. These models had the same variables in block 1 and 2 as those used in the main analyses, both linear and binary logistic, but had three different interaction terms added in block 3: stress*sex, stress*education level, and stress*healthcare professional status. SPSS V.28 was used for analyses (IBM Corp, 2021). An α of 0.05 was used, except when testing for interaction terms, for which we increased the Type I error rate to a less conservative α of 0.10 in order to increase power.

#### 2.3.1 Missing data

Most participants had no missing data (*n* = 111; 95.7%). Five out of 116 participants had data on demographics and changes in work situations, but on none of the other measurements. Because the percentage of missing data on all variables of interest was smaller than 5%, we assumed that imputation would have negligible benefit and proceeded with pairwise deletion (Schafer, 1999).

## 3 Results

### 3.1 Higher-order factorial structure of the brief COPE

The rotated component loadings for the higher-order structure of the Brief COPE are shown in Table 1. The first factor included both social support subscales, venting, and denial; we named this factor “social support and emotional coping”. Note that the self-distraction scale loaded on this subscale but had a higher loading on factor 4 and therefore was not included here. The second factor included the positive reframing, acceptance, and humor scales and seemed to measure “positive cognitive restructuring”. The third factor included the active coping, planning, and suppression of competing activities subscales, and was assumed to measure the higher-order factor of “problem-focused coping”. The fourth factor was made up of restraint, behavioral disengagement, and self-distraction; we named this factor “passive coping”. Suppression of competing activities loads above 0.4 on this factor but loads stronger on the problem-focused coping factor and was included there. The first model resulted in these same four factors in addition to the religion factor. The resulting coping scales showed questionable to good internal reliability; with social support and emotional coping (α = 0.74), positive cognitive restructuring (α = 0.83), problem-focused coping (α = 0.77), and passive coping (α = 0.62).

**Table 1:** Principle component analysis of the brief COPE in Dutch workers (*N* = 111).

COPE subscales			Factors			
	Mean	SD	1	2	3	4
Active coping	6.80	1.08			0.829	
Planning	6.12	1.37			0.863	
Suppression of competing activities	5.07	1.29			0.547	0.477
Restraint	4.87	1.34				0.555
Use of instrumental social support	5.67	1.34	0.656			
Use of emotional social support	5.61	1.40	0.743			
Positive reframing	6.06	1.47		0.703		
Acceptance	5.66	1.43		0.800		
Venting	4.44	1.17	0.774			
Denial	2.82	1.02	0.474			
Behavioral disengagement	2.62	1.01				0.639
Self-Distraction	4.51	1.24	0.429			0.595
Humor	4.70	1.43		0.789		

Rotated component loadings greater than or equal to 0.4 are shown. SD, Standard Deviation.

### 3.2 Characteristics of the worker sample

The majority of the worker sample was female and highly educated, see Table 2. The largest employee group consisted of healthcare professionals (HCP), with 20.7% working as psychiatrist or psychologist, 18% as social worker, 13% as nurse, 6% as other clinical worker. In terms of other professionals, 7% worked in security, 24% in consultancy and management, and 11.2% in secretarial or administrative functions. Compared to other professionals, HCP had a lower age (*t*(114) = 3.163, *p* = 0.002), a higher education level (*χ*^2^*_2_* = 28.531; *p* < 0.001), experienced more changes in work situation (*t*(114) = −3.476, *p* < 0.001), and had higher social support and emotional coping scores (*t*(109) = −3.122, *p* = 0.002). Female workers, when compared to male workers, scored higher in terms of social support and emotional coping (respectively *M* = 19.2, *SD* = 3.30 and *M* = 16.9, *SD* = 3.26; *t*(109) = −3.326, *p* = 0.001) and worked less hours a week (respectively *Mdn* = 30 and *Mdn* = 32;*U* = 1,021, *p* = 0.031). No other differences were found in terms of sample characteristics based on worker type or sex.

**Table 2:** Descriptive characteristics of the worker sample (*N* = 116^a^).

Characteristic (Mean, SD)^a^		Total sample	Healthcare professional (*n* = 67)	Other professional (*n* = 49)
Sex (*n*, %)	*Female*	83 (71.6%)	49 (73.1%)	34 (69.4%)
Age		44.7 (12.2)	41.8 (12.8)	48.8 (10.2)
Education (*n*, %)	*≤ 12 years*	4 (3.4%)	0 (0.0%)	4 (8.2)
	*13–16 years*	26 (22.4%)	5 (7.5%)	21 (42.9%)
	*≥17 years*	86 (74.1%)	62 (92.5%)	24 (49%)
Years in organization^b^ (*Median, IQR*)		6 (6)	5 (5)	6 (4)
Working hours a week (*Median, IQR*)		32 (10)	32 (8)	32 (12)
Changes in work situation		0.48 (0.02)	0.54 (0.03)	0.39 (0.03)
Perceived stress^c^		10.5 (5.7)	11.4 (5.8)	9.3 (5.3)
Social support and emotional coping^c^		18.5 (3.4)	19.4 (3.3)	17.4 (3.3)
Positive cognitive restructuring^c^		16.4 (3.4)	16.2 (3.6)	16.7 (3.2)
Problem-focused coping^c^		18.0 (3.0)	18.3 (3.1)	17.6 (2.7)
Passive coping^c^		12.0 (2.4)	11.7 (2.2)	12.5 (2.6)
Burnout total core symptoms^c^		1.8 (0.4)	1.9 (0.5)	1.8 (0.4)
Aggression total score^c^ (*Median, IQR*)		14 (4)	14 (5)	14 (4)

IQR, interquartile range.

a Unless otherwise specified.

b *N* = 110.

c *N* = 111.

### 3.3 Changes in work situation

**Figure 1:**
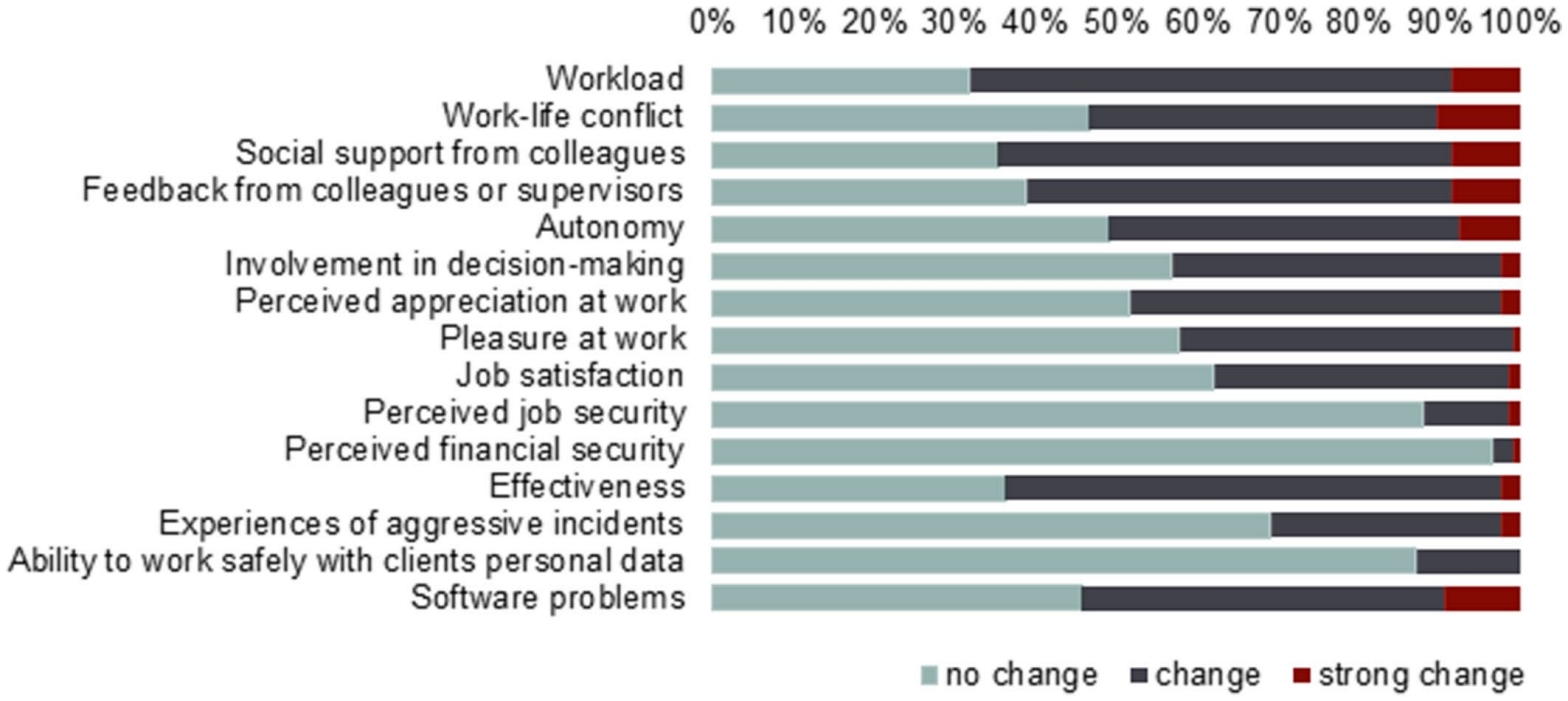
Amount of change in work situation reported since the start of the COVID-19 pandemic by workers (*N* = 116).

Participants reported that in the two weeks before their participation, 59.5% worked at home or at another location than usual for at least 1 h a week, with 34.5% working elsewhere at least half of their contracted worktime. Almost all workers (*n* = 112; 96.6%) reported changes in at least one of the work factors since the start of the COVID-19 crisis. However, on each separate factor a considerable number of workers reported no change (31.9–96.6%); see Figure 1. On average, participants reported increases in terms of workload (*t*(115) = 10.08, *p* < 0.001), work-life conflict (*t*(115) = 7.30, *p* < 0.001), autonomy (*t*(115) = 6.54, *p* < 0.001), experiences of aggressive incidents (*t*(115) = 3.26, *p* = 0.001), and software problems (*t*(115) = 4.92, *p* < 0.001). Participants reported decreases more often than increases in terms of feedback from colleagues or supervisors (*t*(115) = −3.21, *p* = 0.002), involvement in decision making (*t*(115) = −1.98, *p* < 0.001), work pleasure (*t*(115) = −5.22, *p* < 0.001), job satisfaction (*t*(115) = −3.56, *p* < 0.001), effectiveness (*t*(115) = −3.57, *p* < 0.001), and ability to work safely with clients personal data (*t*(115) = −3.52, *p* < 0.001).

### 3.4 Correlations between changes in work situation, stress, coping, burnout symptoms, and aggression

Participants that experienced more changes in work situation overall experienced more stress (*r* = 0.195, *p* = 0.040) and had higher total burnout (*r* = 0.322, *p* < 0.001) and total aggression (*r* = 0.215, *p* = 0.023) scores. The level of experienced changes in the work situation was not correlated with scores on any of the coping scales. Perceived stress was positively and strongly correlated with total burnout (*r* = 0.675, *p* < 0.001) and total aggression (*r* = 0.555, *p* < 0.001). Furthermore, workers with lower stress scores reported higher positive cognitive reframing scores (*r* = −0.336, *p* < 0.001) and lower passive coping scores (*r* = 0.358, *p* < 0.001). Social support and emotional coping scores and problem-focused coping scores were not meaningfully correlated with stress scores (*r* = 0.160, *p* = 0.093; *r* = 0.172, *p* = 0.070).

### 3.5 Associations of stress and coping with burnout symptoms

Hierarchical linear regression models of stress, coping, and their interaction on burnout symptoms were conducted, see Table 3. Although none of the covariates sex, age, education level, and healthcare professional status explained significant variance initially, some were associated with burnout outcomes in the final models; these effects were small. A higher education level was associated with higher total core symptoms (*f^2^* = 0.02), cognitive impairments (*f^2^* = 0.08), and log emotional impairments (*f^2^* = 0.04), females had higher secondary symptom scores than males (*f^2^* = 0.02), age was positively associated with exhaustion symptoms (*f^2^* = 0.03), and HCP had lower cognitive impairment scores than other professionals (*f^2^* = 0.03).

**Table 3:** Linear regression results of the associations of stress and coping on burnout symptoms (N = 111)^a^.

	Burnout symptoms					
	Total core symptoms	Secondary symptoms	Exhaustion	Mental distance	Cognitive impairments	Log emotional impairments
*Main effects*						
Stress	0.73 [0.56–0.91]***	0.63 [0.45–0.81]***	0.68 [0.51–0.87]***	0.47 [0.25–0.68]***	0.63 [0.44–0.81]***	0.59 [0.39–0.78]***
SEC	0.02 [−0.13–0.18]	−0.02 [−0.19–0.15]	−0.05 [−0.22–0.12]	−0.13 [−0.33–0.07]	0.09 [−0.09–0.26]	0.18 [−0.01–0.37]
PCR	−0.09 [−0.23–0.06]	−0.04 [−0.20–0.13]	−0.09 [−0.25–0.07]	−0.04 [−0.22–0.14]	−0.09 [−0.27–0.07]	−0.07 [−0.25–0.09]
PFC	−0.17 [−0.32- -0.01]*	−0.14 [−0.30–0.01]	−0.14 [−0.30–0.02]	−0.13 [−0.32–0.05]	−0.21 [−0.37- -0.04]*	−0.11 [−0.28–0.06]
PC	−0.04 [−0.19–0.12]	0.12 [−0.05–0.29]	−0.03 [−0.20–0.14]	0.04 [−0.17–0.24]	−0.09 [−0.28–0.09]	−0.04 [−0.23–0.14]
Interactions						
Stress*SEC	–	0.05 [−0.11–0.22]	–	–	–	–
Stress*PCR	–	−0.25 [−0.41- -0.10]**	–	–	–	–
Stress*PFC	–	−0.07 [−0.23–0.10]	–	–	–	–
Stress*PC	–	−0.09 [−0.25–0.06]	–	–	–	–
*F* change*; R*^2^ total^b^						
Step 1	0.69; −	1.38; −	0.87; −	0.98; −	1.60; −	0.80; −
Step 2	20.62***; 0.47	15.19***; 0.41	16.72***; 0.42	6.27***; 0.20	12.89***; 0.37	11.27***; 0.32
Step 3	0.47; −	3.10*; 0.45	0.77; −	1.15; −	1.06; −	0.83; −

PC, passive coping; PCR, positive cognitive restructuring; PFC, problem-focused coping; SEC, social support and emotional coping.

a Standardized beta coefficients [95% confidence interval] based on the final improving model are presented after controlling for workers’ age, sex, education level and healthcare professional status in step 1. Significant coefficients and model improvements are presented in bold.

b Adjusted *R*^2^for the total model is presented.

*p < 0.05; **p < 0.01; **p < 0.001.

Perceived stress was positively associated with all burnout outcomes, the effect sizes were medium to large: total burnout (*f^2^* = 0.34), secondary symptoms (*f^2^* = 0.24), exhaustion (*f^2^* = 0.30), mental distance (*f^2^* = 0.14), cognitive impairments (*f^2^* = 0.26), and log emotional impairments (*f^2^* = 0.23). Problem-focused coping was negatively associated with total core symptoms (*f^2^* = 0.02) and with cognitive impairment (*f^2^* = 0.04); i.e., individuals with higher problem-focused coping scores had lower total core symptom and cognitive impairment scores. These effect sizes are considered small. Problem-focused coping had negative standardized beta coefficients on all burnout outcomes, but for the other outcomes the association was not statistically significant.

**Figure 2:**
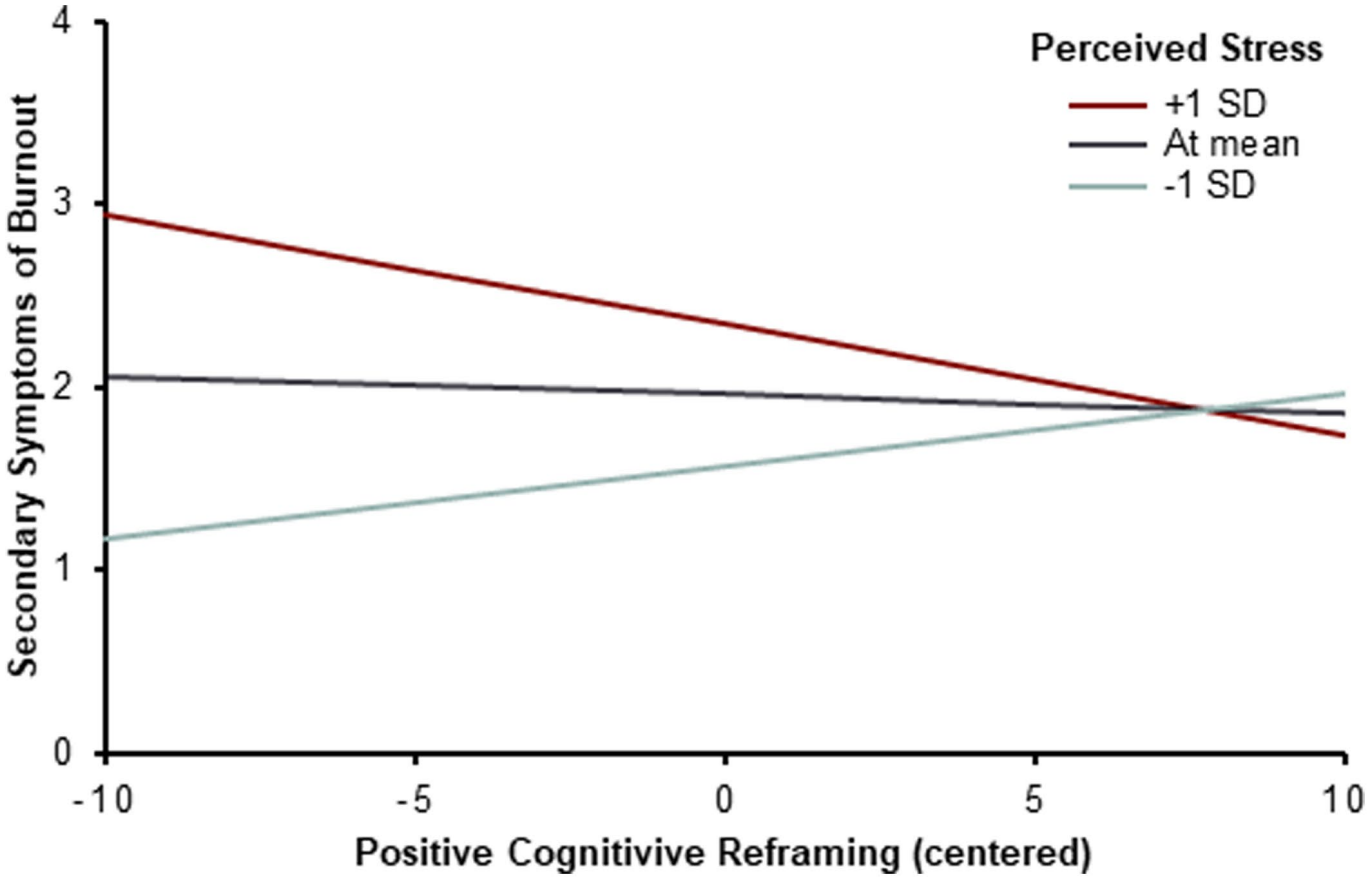
Interaction effect of perceived stress and positive cognitive reframing on secondary burnout symptoms (*N* = 111). Based on *post-hoc* simple slope analyses positive cognitive reframing is associated with secondary burnout symptoms in the following ways: **(A)** positively associated at 1 standard deviation below the mean stress scores (*b* = 0.04, *p* = 0.016), **(B)** not associated at the mean stress scores (*b* = −0.01, *p* = 0.658), **(C)** negatively associated at 1 SD above the mean stress scores (*b* = −0.06, *p* = 0.020).

Testing potential interaction effects between stress and coping, we found only evidence for an interaction in the association with secondary burnout symptoms. Perceived stress interacted with positive cognitive restructuring to influence secondary burnout symptoms (*f^2^* = 0.06); the effect was small. For those with higher perceived stress scores (1 *SD* above the sample mean), more positive cognitive restructuring was related to less secondary symptoms of burnout, see Figure 2. For those with a mean stress score, positive cognitive restructuring was not related to secondary burnout symptoms. For those with lower perceived stress scores (1 *SD* below the sample mean), more positive cognitive restructuring was related to more secondary burnout symptoms.

### 3.6 Associations of stress and coping with symptoms of aggression

Hierarchical binary logistic regression models were conducted of stress, coping, and their interaction, on aggression symptoms, dichotomized at the 80th percentile; see Table 4. Looking at the covariates, older participants were more likely to report lower hostility scores; which remained significant once corrected for stress and coping (*OR* = 0.93, 95% CI [0.88–0.99], *p* = 0.24). Higher levels of perceived stress were associated with a higher likelihood to report all types of aggression: total aggression, verbal aggression, anger, and hostility. These effects were very small. Positive cognitive restructuring was negatively associated with total aggression and hostility; again, the effect was very small. We found no indication of other interaction effects of stress and coping for any of the aggression outcomes.

**Table 4:** Logistic regression results of the associations of stress and coping on aggression (*N* = 111)^a^.

	Aggression			
	Total	Verbal	Anger	Hostility
*Main effects*				
Stress	1.26 (1.10–1.45)***	1.16 (1.04–1.29)***	1.23 (1.09–1.4)***	1.23 (1.07–1.42)**
SEC	0.89 (0.73–1.08)	1.06 (0.90–1.25)	0.97 (0.82–1.16)	1.05 (0.84–1.3)
PCR	0.81 (0.65–1.00)*	0.97 (0.83–1.14)	0.98 (0.82–1.17)	0.74 (0.59–0.94)*
PFC	1.17 (0.92–1.48)	0.88 (0.72–1.06)	1.11 (0.91–1.37)	1.17 (0.9–1.51)
PC	0.94 (0.71–1.25)	1.02 (0.81–1.29)	0.81 (0.62–1.05)	1.29 (0.95–1.76)
*Interactions*				
Stress*SEC	–	**–**	**–**	**–**
Stress*PCR	–	**–**	**–**	**–**
Stress*PFC	–	**–**	**–**	**–**
Stress*PC	–	–	–	–
*χ*^2^ step; *R*^2^ total^b^				
Step 1	6.22; −	0.75; −	1.05; −	12.43*; 0.17
Step 2	30.10***; 0.44	12.94*; 0.18	17.40**; 0.24	36.99***; 0.56
Step 3	3.72; −	2.26; −	5.59; −	3.48; −

PC, passive coping; PCR, positive cognitive restructuring; PFC, problem-focused coping; SEC, social support and emotional coping.

^a^Odds ratios and 95% confidence intervals based on the final improving model are presented after controlling for workers’ age, sex, education level, and healthcare professional status in step 1. Significant coefficients and model improvements are presented in bold.

b Nagelkerke *R*^2^for the total model is presented.

*p < 0.05; **p < 0.01; ***p < 0.001.

### 3.7 Sensitivity analyses

Interaction effects between stress and sex, stress and education level, and stress and healthcare professional status were explored; we found two effects of interest. First, in the interaction block for the cognitive impairment burnout symptoms (*F* change ^block 3^_3, 98_ = 2.29, *p* = 0.083, *R*^2^change = 0.038) there was an interaction effect of stress by sex. The interaction term indicated that the association between stress and cognitive impairment was stronger in females than in males (*β* = 0.43, *p* = 0.016; *f^2^ = 0.03*); the effect was small. Also, in the interaction block for the total aggression outcome (*χ*^2^_3_ = 6.65, *p* = 0.083, Nagelkerke *R*^2^ total = 0.51) there was an interaction effect of stress by sex. The interaction term indicated that the association between stress and total aggression was stronger in females than males (*OR* = 1.38 [95% CI = 1.01–1.87], *p* = 0.041); the effect was very small.

## 4 Discussion

The current study investigated associations between stress, coping, burnout, and aggression in a sample of forensic mental healthcare workers. These associations were studied during the first months of the COVID-19 pandemic, a stressful period during which workers may have been at increased risk of experiencing burnout symptoms and aggression. We found that workers who experienced more changes in their work situation during COVID-19 reported more stress, burnout symptoms, and aggression. In line with our expectations, we also found that higher amounts of perceived stress were associated with more burnout symptoms and aggression. In this worker sample, problem-focused coping was associated with less overall burnout and cognitive impairment symptoms and positive cognitive restructuring was associated with less overall aggression and hostility. Positive cognitive restructuring was also negatively related to secondary symptoms of burnout for workers that experienced increased amounts of stress, but not in workers with average or low stress.

Our findings that stressors, in the form of changes in work situation, and perceived stress are related to adverse outcomes, in the forms of burnout symptoms and aggression, are in line with the job-demands and resources theory and the GST (Agnew, 1992; Bakker and Demerouti, 2014), and present a possible explanation for the increases in adverse health-related outcomes that were reported among healthcare workers during COVID-19 in previous studies (Giusti et al., 2020; Hacimusalar et al., 2020; Denning et al., 2021; Ibar et al., 2021; Marcil et al., 2022). Changes in work situation may have caused extra stress, either perceived, biologically, or both, and thereby influenced stress-related adverse outcomes. The causality of these associations merits further study. Furthermore, it would be interesting to investigate which work stressors (e.g., workload, perceived effectiveness, or autonomy) are particularly important in causing stress and stress-related outcomes, so that these can potentially be targeted by preventive interventions, in case they are controllable stressors.

Our findings that problem-focused coping and positive cognitive restructuring were related to less adverse outcomes in workers are in line with our expectations and some earlier findings. Shin et al. (2014) found in their meta-analysis that more problem-focused coping was related to less symptoms of burnout and Gurvich et al. (2021) found that positive cognitive restructuring strategies were related to better mental health. We could not confirm that emotion-focused coping was related to more burnout (Shin et al., 2014; Mariani et al., 2020), nor that active coping was related to less aggression and passive coping to more aggression (Hu and Sun, 2023). These discrepancies may have been influenced by the (dis)ability to implement certain coping strategies in the specific context of a pandemic (Lazarus and Folkman, 1984). For example, an individual might have been able to use problem-focused coping and positive cognitive restructuring relatively independently, whereas social support and emotional coping is more dependent on interaction with other people. Others may have been less available physically or emotionally or may have been less able to offer resources due to their own struggles during the pandemic. Some forms of passive coping may also have been more difficult to implement, because so many activities had been canceled during the pandemic and therefore could not be used for self-distraction. Considering the average coping scores presented in Table 2 and taking into account the number of items per factor, our sample did indeed report more use of positive cognitive reframing and problem-focused coping (both 6 items), than social support and emotional coping (10 items), and passive coping (8 items).

Due to the cross-sectional design of our study, we are not able to draw firm conclusions concerning the causal relationships underlying our results and may only cautiously speculate on potential mechanisms. First, we speculate that the negative associations of problem-focused coping with burnout symptoms and cognitive impairment may be bidirectional. Individuals that apply problem-focused coping strategies more effectively, may be more able to positively influence situations that require cognitive performance, for example by planning when to work on cognitively demanding tasks or writing down next steps of action. In reverse, more cognitive impairment due to burnout might negatively affect the ability to apply problem-focused coping, which in turn may increase burnout symptoms. Cognitive impairments like low concentration, attention and memory might make it hard to formulate a plan, or to put it into action. More research on the associations between cognitive functioning and coping might shed light on the causal direction of these associations. Second, we found that positive cognitive restructuring was negatively related to secondary symptoms of burnout only in workers that experienced increased amounts of stress and therefore we speculate that a certain level of stress, and in effect a sense of urgency, must be experienced in order for this way of coping to influence well-being. For those who experience low or moderate amounts of stress, cognitive reframing strategies may not be helpful or even needed. Third, we speculate that positive cognitive reframing may be particularly helpful for combating cognitive forms of aggression. In our study, positive cognitive reframing was associated with overall aggression and hostility, yet not with verbal aggression or anger, forms that may be more related to emotions than cognitions.

The four higher-order factors of coping that we found are mostly in line with previous research. That is, although there is no clear consent on the core higher-order categories of coping, similar categories have often been distinguished in other studies (e.g., see Skinner et al. for a comprehensive review up to 2003). Our results point to the same four higher-order factors distinguished in Carver et al.’s original analyses of the COPE Carver et al. (1989), although the underlying coping strategies differed somewhat. In study 1, Carver et al. likewise concluded that religion did not fit into any higher-order factor. Our findings are also in line with the five core higher-order factors based on Skinner et al.’s (2003) large systematic review, although support seeking loaded with emotional coping on the same factor, and distraction did not present as a separate factor. Our findings raise the question whether support seeking and emotional coping should be considered as one larger factor and the same goes for avoidance and self-distraction; that would be in line with Carver et al.’s (1989) findings on higher-order coping factors as well. Furthermore, our results point to a particular problematic issue in the coping literature (Skinner et al., 2003; Folkman and Moskowitz, 2004; Compas et al., 2017), where researchers use similar terms for higher-order coping categories but use different definitions or operationalization. This may lead to inconsistencies in findings and miscommunication. To prevent these issues, clearly defining higher-order coping factors is an important avenue for future research.

## 5 Strengths and limitations

The current study adds to the knowledge on occupational and health psychology, and to the broader research on the structure of coping. We assessed various burnout symptoms and aggression using well-validated questionnaires. However, our relatively small sample size may have hampered the detection of potential interaction effects, notwithstanding the use of rather complex hierarchical models. Second, we used self-reported measures, which may have led to response bias. However, perceived stress and coping are still best measured by self-report, particularly in a worker population, where most individuals experience subclinical levels of burnout and aggression. Third, because our study design was cross-sectional, temporal relations could not be assessed and conclusions on causality cannot be drawn from our results. Finally, our results may be affected by selection effects, where workers with very high levels of burnout did not participate. One might assume that this selection may lead to underestimation of the effects of stress on burnout symptoms and aggression, but it is impossible to infer from our results or to rule out selection bias.

## 6 Conclusions and implications

In this worker sample, that reported during COVID-19, problem-focused coping and positive cognitive restructuring were related to less burnout symptoms and aggression. Problem-focused coping may protect against burnout symptoms in particular, while positive cognitive restructuring may protect against aggressive cognitions in particular. The benefit of these two ways of coping is that they are mainly individual activities and therefore they may be helpful in intervention programs aimed at stressed workers, without the workers having to rely on others, as is the case with support-seeking types of coping. Perceived stress was strongly and consistently related to all burnout symptom dimensions and also consistently related to aggression, making it a prime target for monitoring worker’s well-being. Because sensitivity analyses showed that associations between stress and, respectively, cognitive impairment and overall aggression, were stronger in females than in males, preventive interventions may initially focus on female sex as a risk factor. More research on training and applying these protective coping strategies is needed in an effort toward promoting workers’ well-being. Work burnout may have serious consequences, for individual health of the workers themselves, but also for organizational and occupational outcomes such as diminished job performance, absenteeism and high turnover. By that, burnout and aggression by staff may also affect the quality of services provided to the population of patients in forensic health care. There is a clear need to acknowledge workers’ well-being and, where needed, to invest in programs that promote stress management training, and on-demand professional counseling.

## Data availability statement

The raw data supporting the conclusions of this article will be made available by the authors, without undue reservation.

## Ethics statement

The studies involving humans were approved by the institutional review board of Dimence Group (CWO-062020PSFB). The studies were conducted in accordance with the local legislation and institutional requirements. The participants provided their written informed consent to participate in this study.

## Author contributions

PS: Conceptualization, Data curation, Formal analysis, Investigation, Methodology, Project administration, Resources, Validation, Visualization, Writing – original draft preparation. FB: Data curation, Conceptualization, Investigation, Project administration, Resources, Writing – review & editing. YB: Conceptualization, Funding acquisition, Supervision, Writing – review & editing. WH: Funding acquisition, Supervision, Writing – review & editing. SR: Conceptualization, Funding acquisition, Methodology, Supervision, Writing – review & editing.
